# Maternal Deprivation Exacerbates the Response to a High Fat Diet in a Sexually Dimorphic Manner

**DOI:** 10.1371/journal.pone.0048915

**Published:** 2012-11-07

**Authors:** Virginia Mela, Álvaro Llorente-Berzal, Francisca Díaz, Jesús Argente, María-Paz Viveros, Julie A. Chowen

**Affiliations:** 1 Department of Physiology (Animal Physiology II), Faculty of Biology, Universidad Complutense, Instituto de Investigación Sanitaria del Hospital Clínico San Carlos, Madrid, Spain; 2 Department of Endocrinology, Hospital Infantil Universitario Niño Jesús, Instituto de Investigación Biomédica Princesa, Madrid, Spain; 3 Department of Pediatrics, Universidad Autónoma de Madrid, Madrid, Spain; 4 CIBER de Fisiopatología de Obesidad y Nutrición, Instituto Carlos III, Madrid, Spain; University of Cordoba, Spain

## Abstract

Maternal deprivation (MD) during neonatal life has diverse long-term effects, including affectation of metabolism. Indeed, MD for 24 hours during the neonatal period reduces body weight throughout life when the animals are maintained on a normal diet. However, little information is available regarding how this early stress affects the response to increased metabolic challenges during postnatal life. We hypothesized that MD modifies the response to a high fat diet (HFD) and that this response differs between males and females. To address this question, both male and female Wistar rats were maternally deprived for 24 hours starting on the morning of postnatal day (PND) 9. Upon weaning on PND22 half of each group received a control diet (CD) and the other half HFD. MD rats of both sexes had significantly reduced accumulated food intake and weight gain compared to controls when raised on the CD. In contrast, when maintained on a HFD energy intake and weight gain did not differ between control and MD rats of either sex. However, high fat intake induced hyperleptinemia in MD rats as early as PND35, but not until PND85 in control males and control females did not become hyperleptinemic on the HFD even at PND102. High fat intake stimulated hypothalamic inflammatory markers in both male and female rats that had been exposed to MD, but not in controls. Reduced insulin sensitivity was observed only in MD males on the HFD. These results indicate that MD modifies the metabolic response to HFD intake, with this response being different between males and females. Thus, the development of obesity and secondary complications in response to high fat intake depends on numerous factors.

## Introduction

Studies investigating the long-term effects of the early developmental environment on health and well-being have increased dramatically in recent years. As a result the relationship between early events such as stress, illness, infection, nutrition or pharmacological treatments and the propensity towards a vast number of diseases has become apparent [1]–[5]. Moreover, these long-term responses to developmental perturbations often differ between males and females, which may partially explain the difference between the sexes in the prevalence of some diseases [1], [5]–[10].

The dramatic rise in obesity during the past decades has been largely attributed to changes in lifestyle, with less physical activity and increased energy intake contributing greatly to this phenomenon. In addition, early environmental influences including both dietary modifications and diverse stresses can affect the response to a poor diet in later life, thus modulating the possibility of becoming obese and with many of these long-term effects being sexually dimorphic [10]–[14]. Maternal deprivation (MD), which in some cases induces extended food restriction, as well as dehydration and thermal and psychological stress, can have long-lasting effects on diverse systems and functions, including metabolism [9], [15]–[19]. Indeed, MD for 24 hours decreases body weight and circulating leptin levels in rats on a normal laboratory diet, with this effect being both age and sex dependent [6], [16]. However, little information is available regarding how these animals respond to metabolic challenges later in life.

Increased intake of high fat content foods is indicated as one of the main causes of obesity, as well as the secondary complications associated with weight gain. High fat intake is reported to induce hypothalamic inflammation and this inflammation is thought to be involved in the development of insulin and leptin resistance, resulting in further weight gain and secondary complications [20]–[22]. Thus, in this study we aimed to determine the weight gain and metabolic responses of MD rats to a high fat diet (HFD) introduced at the time of weaning, comparing the responses between males and females. In addition, association of weight gain with hypothalamic inflammatory processes was analyzed.

## Materials and Methods

### Ethics Statement

These studies were approved by the ethics committee of the Universidad Complutense de Madrid and complied with the Royal Decree 1201/2005 (BOE n° 252) pertaining to the protection of experimental animals and with the European Communities Council Directive (86/609/EEC). Special care was taken to reduce animal suffering and to use the minimum number of animals for all studies.

**Figure 1:**
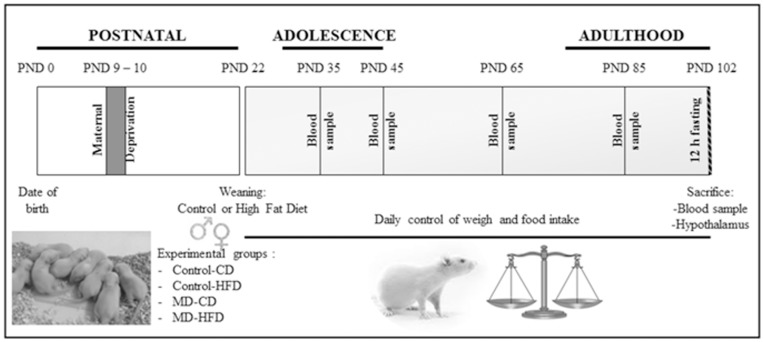
Experimental design. Animals were deprived from their mother during 24h at postnatal day (PND) 9. From weaning (PND 22) and throughout the study rats received either a control or a high fat diet. Body weight and food intake were monitored daily. At PNDs 35, 45, 65 and 85 blood samples were obtained from the tail vein. At PND 102 animals were sacrificed after a 12 h fasting period, trunk blood was collected and the hypothalamus was dissected-out and rapidly frozen.

### Animals

Adult Wistar rats were purchased from Harlan Interfauna Ibérica S.A. (Barcelona, Spain) and allowed to acclimate for 2 weeks before mating. One male was placed in a cage with two females for 10 days. On the day of birth litters were culled to eight pups per dam (four males and four females), with no cross-fostering employed. In all experimental groups at least three different litters were used to reduce the litter effect, with a total of 12 rats in each experimental group.

Rats were maintained at a constant temperature (22±1°C) and humidity (50±2%) in a reversed 12-h light-dark cycle (red light on at 0∶800 and white light on at 20∶00). Pregnant rats were given free access to rat chow (commercial diet for rodents A03; Safe, Augy, France) and water.

### Maternal Deprivation

Maternal deprivation was performed as previously described [6]. Briefly, beginning at 09∶00 on postnatal day (PND) 9, mothers from the deprived group were removed and placed in a cage beside the home cage in the same room. On PND10 at 09∶00, mothers were returned to the cage of their respective litters. Mothers of the control litters were left undisturbed. All rats were left undisturbed until weaning at PND22 at which time 4 rats of the same sex and experimental group were placed in each cage. Half of each group received either a HFD (45% fat; D12451, Research Diets, New Brunswick, NJ, USA) or a control diet CD (10% fat; D12450B, Research Diets) and allowed to eat *ad libitum.* Body weight and food intake were monitored daily until PND101. Food intake was measured by placing a known amount of food in each cage and measuring the remaining amount at the same time the next day. This was then divided by the number of animals/cage, with statistics being performed using this value (n  =  the number of cages/experimental group).

**Figure 2:**
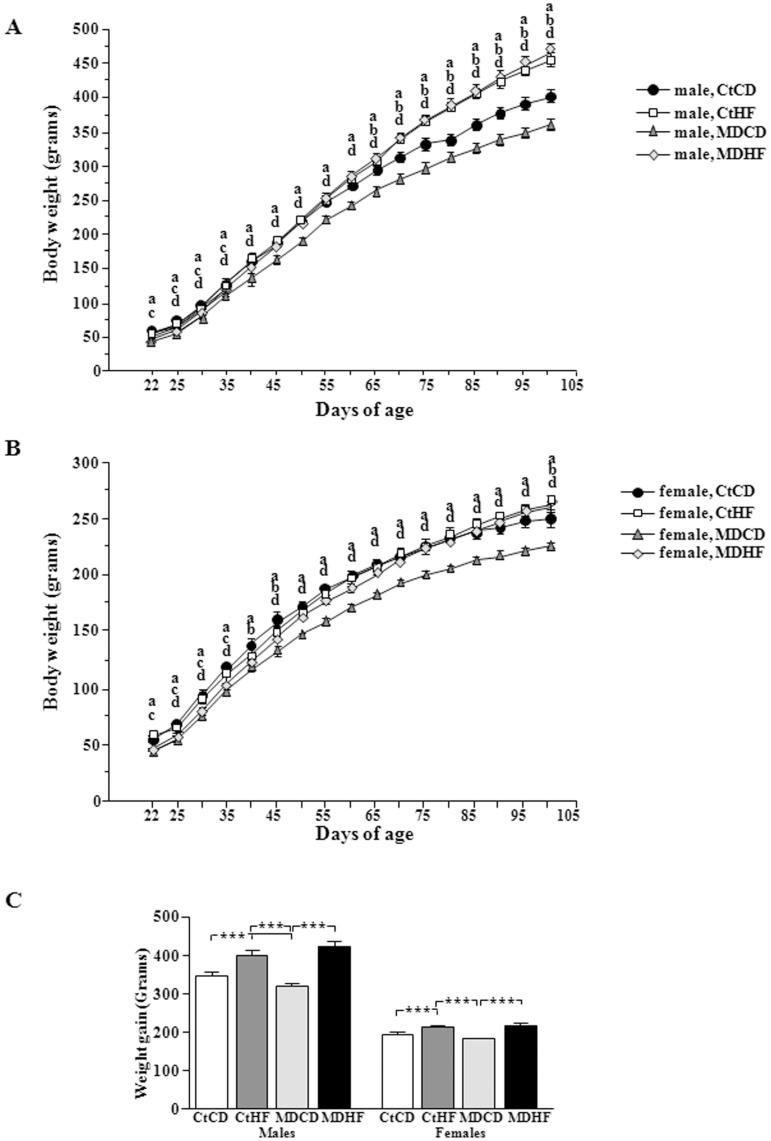
Body weight gain from the time of weaning (day 22 of age) until day 101 of life in males (A) and females (B). Total weight gain from the time of weaning until day 101 of life (C). CtCD: controls on a control diet; CtHF: controls on a high fat diet from weaning onward; MDCD: maternally deprived rats on a control diet; MDHF: maternally deprived rats on a HFD from weaning onward. Significant differences: a: CtCD vs MDCD; b: CtCD vs CtHF; c: CtHF vs MDHF; d: MDCD vs MDHF. ***  =  ANOVA p<0.0001.

This resulted in the following experimental groups in both sexes: control, CD (CtCD), control HFD (CtHF), maternally deprived CD (MDCD), and maternally deprived HFD (MDHF). Post-pubertal female rats were sacrificed randomly throughout the estrous cycle at the stipulated ages.

Non-fasting blood samples were collected from the tail vein at PNDs 35, 45, 65 and 85. On PND102 all rats were sacrificed after a 12h fast by rapid decapitation and trunk blood was collected in tubes containing EDTA (0.5 M) and rapidly placed on ice. The blood was centrifuged (3000 rpm for 15 min) and the plasma collected and stored at −80° until processed. The brain was rapidly removed and the hypothalamus dissected. It was then frozen in liquid nitrogen and stored at −80° until processed.

A schematic representation of the experimental design and tissue sample collection is shown in Figure 1.

**Figure 3:**
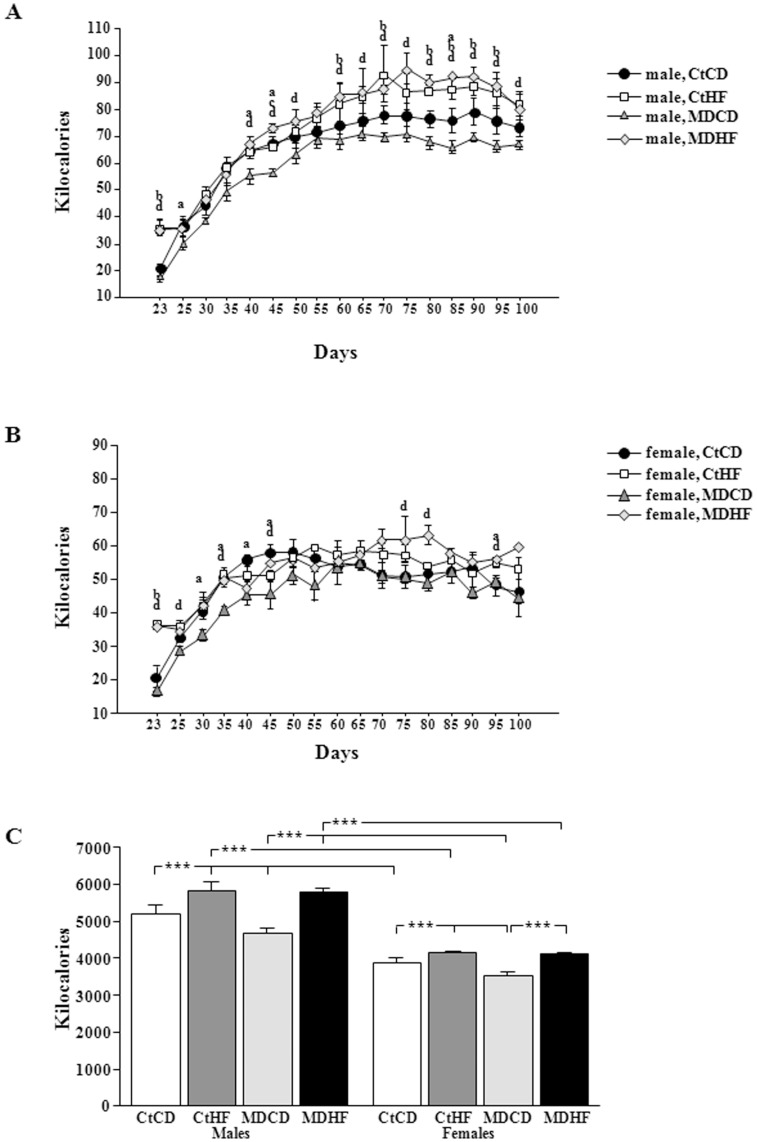
Kilocalorie intake from the time of weaning (day 22 of age) until day 101 of life in males (A) and females (B). Total kilocalorie intake throughout the study (C). CtCD: controls on a control diet; CtHF: controls on a high fat diet from weaning onward; MDCD: maternally deprived rats on a control diet; MDHF: maternally deprived rats on a HFD from weaning onward. Significant differences: a: CtCD vs MDCD; b: CtCD vs CtHF; c: CtHF vs MDHF; d: MDCD vs MDHF. ***  =  ANOVA p<0.0001.

### Plasma Insulin, Leptin, Glucose and Triglyceride Measurements

Plasma leptin levels were measured by using B-bridge mouse/rat leptin ELISA kits (Cupertino, CA, USA) and insulin with Mercodia rat insulin ELISA kits (Uppsala, Sweden) following the manufacturers’ instructions. For insulin the sensitivity of the assay was 0.07 µg/ml with an inter- and intra-assay variation of 3.3% and 2%, respectively. The assay sensitivity for the leptin assay was 0.5 ng/ml with an inter-assay variation 6.5% and intra-assay variation of 3.7%. All samples were run in duplicate.

Plasma glucose and total triglycerides were measured by using commercial kits (RANDOX, Antrin, UK) according to the manufacturer’s instructions. Briefly, to measure triglycerides plasma was incubated with the commercial solution containing lipoprotein lipase, glycerol kinase, glycerol-3-oxidase, peroxidase, 4 aminophenazone and ATP for 5 minutes at 37°C under agitation. The resulting color was detected at 500 nm in a spectrophotometer and compared to a standard curve. Glucose levels were determined by incubating the samples with the commercial solution containing peroxidase, 4-aminofenazone and glucose oxidase for 10 min at 37°C under agitation and the results determine as described above.

**Figure 4:**
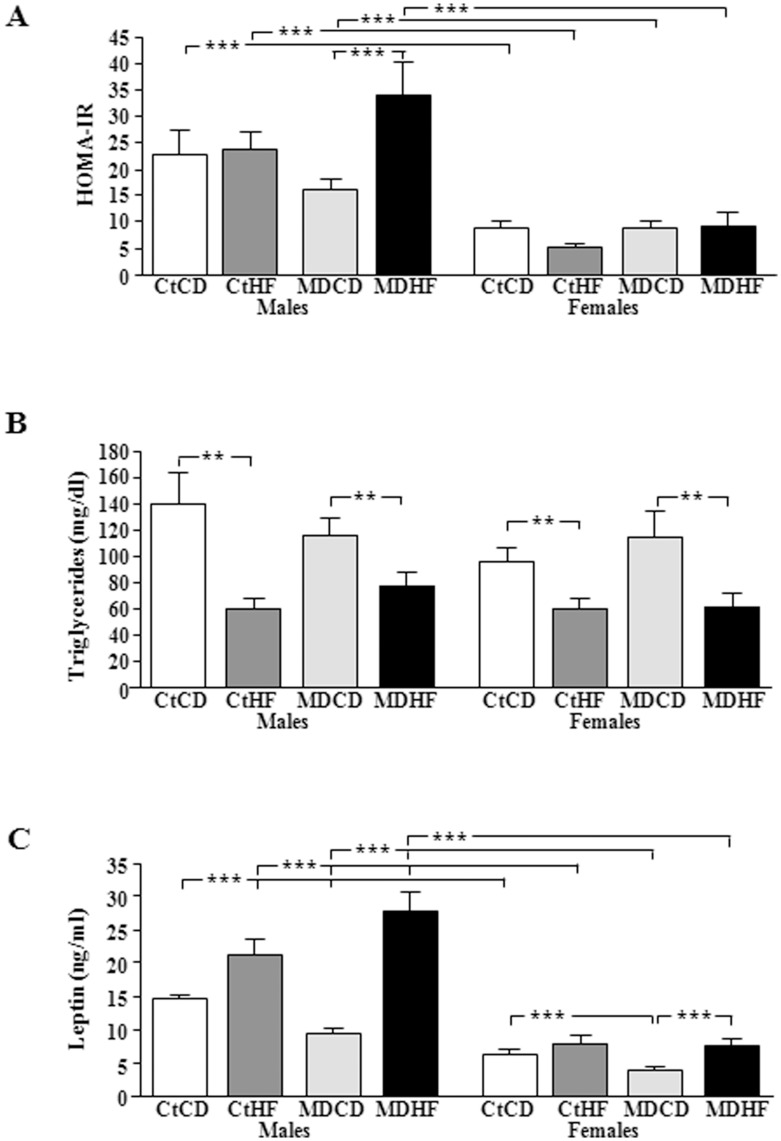
Mean HOMA index (A) and serum triglyceride (B) and leptin (C) levels at the time of sacrifice. CtCD: controls on a control diet; CtHF: controls on a high fat diet from weaning onward; MDCD: maternally deprived rats on a control diet; MDHF: maternally deprived rats on a HFD from weaning onward. ***  =  ANOVA p<0.0001., **  =  ANOVA p<0.0005. NS  =  not significant.

The homeostasis model assessment (HOMA) index was used to access insulin sensitivity at PND102. This was calculated by using the formula: HOMA-IR  =  [Glucose (mmol/l) x insulin (µU/ml)]/22.5.

### Quantitative Real-time PCR

Total RNA was extracted from each hypothalamus with TRIzol® Reagent (Invitrogen). High Capacity cDNA Reverse Transcription kits (Applied Biosystems, Foster City, CA) were used according to the manufacturer’s protocol on a Peltier thermal Cycler Tetrad2 (BioRad) to transcribe 2 µg total RNA isolated from each individual hypothalamus. Amplification of the cDNA template was performed with an ABI PRISM 7900HT Sequence Detection System (Applied Biosystems) using TaqMan Universal PCR Master Mix (Applied Biosystems) and TaqMan Gene Expression Assay kits for each detected gene (Applied Biosystems). The commercial reference for each predesigned expression assay is as follows for each gene measured in the hypothalamus: neuropeptide Y (NPY, Rn01410145), proopiomelanocortin (POMC; Rn00595020), Agouti-related peptide (AgRP; Rn014311703), cocaine and amphetamine-regulated transcript (CART; Rn00567382), interleukin (IL)-1β (Rn00580432), IL-6 (Rn01410330), TNF-α (Rn01525859), leptin receptor (LepR; Rn01433250), suppressor of cytokine signaling 3 (SOCS3; Rn00585674) and glial fibrillary acidic protein (GFAP; Rn00566603). Results were normalized to 18S (Rn01428915) mRNA levels in all samples. According to manufacturer’s guidelines, the ΔΔCT method was used for relative quantification. Statistics were performed using ΔΔCT values.

**Table 1:** Serum levels of glucose (n = 10), insulin (n = 5), triglycerides (n = 11–12) and leptin (n = 12) at 35, 45, 65 and 85 days of age.

	Age	Males				Females			
		CtCD	CtHF	MDCD	MDHF	CtCD	CtHF	MDCD	MDHF
**Glycemia** **(mg/dl)**	35	147.1±9.8	152.1±10.1	150.5±9.5	140.9±13.4	138.3±9.1	124.8±9.2 **d**	105.6±10.1**a,da,d**	154.2±13.1 **b,c**
	45	160.0±14.3	157.9±9.1	151.0±9.8	146.2±7.7	134.8±13.6	148.1±16.7	142.9±12.3	136.2±6.4
	65	137.3±12.1	148.5±6.3	146.6±7.4	144.1±4.8	156.5±5.4	147.8±9.8	144.8±13.0	131.9±8.4
	85	136.0±9.8	146.6±5.5	122.4±8.2	117.3±9.3	129.5±5.4	130.4±6.5 **d**	126.4±6.1	124.1±8.1
**Insulin** **(ng/ml)**	35	2.0±0.3	2.0±0.3	1.9±0.3	1.5±0.3	2.5±0.4	1.7±0.5	1.2±0.1	1.8±0.4
	45	7.9±0.5	4.0±0.8 **a**	5.3±0.6 **a**	5.2±1.2 **a**	3.7±0.5 **d**	2.9±0.2	3.2±0.4 **d**	3.1±0.7 **d**
	65	7.8±0.3	5.3±0.8 **a**	6.3±0.9	5.4±0.8	4.4±0.4 **d**	2.5±0.3 **a,d**	3.3±0.4 **d**	2.0±0.4 **b,d**
	85	12.7±1.1	10.6±1.9	7.6±1.3 **a**	8.3±2.1	4.3±0.9 **d**	2.7±0.4 **d**	2.9±0.5 **d**	2.7±0.4 **d**
**Triglyce-rides** **(mg/dl)**	35	135.1±13.2	103.9±24.7	93.1±24.5	93.6±20.5	112.3±16.4	68.0±16.3	59.3±14.8 **a**	76.9±13.5
	45	143.4±14.8	140.9±29.8	172.0±8.6	144.4±28.9	101.9±12.0	73.1±12.5 **d**	100.1±16.4 **d**	78.0±10.5 **d**
	65	157.1±15.2	215.8±43.0	137.5±17.5	150.1±28.5	169.8±21.3	224.5±40.6	205.1±40.2	97.9±23.4
	85	162.1±21.2	301.7±39.2	197.0±38.7	157.5±29.0	177.7±20.4	135.2±16.5 **d**	190.9±45.2	151.4±35.7
**Leptin** **(ng/ml)**	35	6.5±0.4	6.5±0.8	4.0±0.4 **a**	6.1±0.8 **b**	7.2±0.6	6.0±1.0	2.6±0.5 **a**	4.8±0.7 **b**
	45	8.0±0.7	8.3±0.7	4.0±0.4 **a**	9.5±0.9 **b**	8.8±0.7	8.2±1.0	4.3±0.5 **a**	6.5±0.7 **b,d**
	65	15.6±0.7	17.3±1.3	11.3±0.9 **a**	19.2±2.5 **b**	12.7±0.8 **d**	11.3±1.3 **d**	8.5±0.9 **a,d**	11.5±1.4 **d**
	85	21.5±1.3	28.8±2.4 **a**	14.0±1.4 **a**	34.4±3.9 **a,b**	12.6±0.7 **d**	14.6±1.7 **d**	9.01±0.6 **a,d**	14.7±1.0 **b,d**

CtCD: controls on a control diet; CtHF: controls on a high fat diet from weaning onward; MDCD: maternally deprived rats on a control diet; MDHF: maternally deprived rats on a HFD from weaning onward. a: different from CtCD of same sex; b: different from MDCD of same sex; c: different from CtHF of same sex; d: different from males of same treatment.

### Statistical Analysis

Body weight gain and food intake were analyzed by three-way analysis of variance (ANOVA) with the factors being sex (males and females), MD (maternal non deprived and deprived rats) and diet (control or HF), with repeated measures. Three-way ANOVA was used to analyze results when tissue from males and females were processed simultaneously (hormone assays), followed by two-way and one-way ANOVAs when appropriate. Two-way ANOVAs were employed when samples from males and females were processed separately (*e.g*., RT-PCR). Scheffe’s F test was used for post-hoc comparisons. Simple regression analysis was performed to determine the relationship between circulating metabolic factors and body weight and weight gain. The level of significance was chosen as p<0.05. All results are reported as mean ± SEM. In the figures only physiologically relevant comparisons are demonstrated. Although weight gain and food intake over time were analyzed simultaneously in both sexes, the results are represented in separate figures to facilitate interpretation.

## Results

### Weight Gain and Food Intake

Weight gain over time depended on sex (p<0.0001) and diet (p<0.0001), with interactions between sex and diet (p<0.0001) and MD and diet (p<0.0001). In males weight gain was affected by MD (p<0.005) and diet (p<0.0001) with an interaction between these two factors (p<0.01; Fig. 2A). Similar effects were found in females [MD (p<0.0001), diet (p<0.005), MD x diet (p<0.01; Fig. 2B)].

This resulted in total weight gain also being affected by sex (p<0.0001) and diet (p<0.0001) with the effect of diet being influenced by sex (p<0.0001) and MD (p<0.005). Males gained more weight than females in all corresponding groups (p<0.0001; Fig. 2C). After weaning, male MD rats gained less weight than their controls, but MD females did not. All rats consuming HFD gained more weight than their controls (Fig. 2C). Mean weight gain was increased by HFD by approximately 16% in control males, 33% in MD males, 10% in control females and 20% in MD females compared to their appropriate controls on a normal diet.

The final weights at sacrifice were affected by sex (p<0.0002), MD (p<0.05) and diet (p<0.0001) with interactions between sex and diet (p<0.0001) and MD and diet (p<0.002). Although control females gained significantly more weight on the HFD than on the CD, this was not reflected in their final weight. Likewise, MD did not affect post-weaning weight gain in females, but the final weight remained less due to the difference in starting weight when they were weaned. Both sex (p<0.0001) and diet (p<0.02) affected Kcal intake over time. In males (Fig. 3A) MD rats ate less than controls with this being significant at specific time-points. During the first day on the HFD (PND23) Ct and MD rats of both sexes ate considerably more than their controls (Figs. 3A and 3B). Until approximately PND45 no difference in Kcal intake was found between Ct and MD rats on the HFD, but afterwards MD males ate significantly more Kcal than their controls. In control males the increase in Kcal intake on the HFD diet occurred at a later age. On the control diet, MD females ate significantly less than their controls at various time-points. Subsequent to the first day, HFD did not affect Kcal intake in control females, whereas in MD females it increased Kcal intake compared to their controls at specific time-points.

Total accumulated Kcal intake throughout the study (Fig. 3C) was influenced by sex (p<0.0001), MD (p<0.05) and type of diet (p<0.0001). Males ate more than females in all corresponding groups (p<0.0001). On a normal diet MD males (p<0.0005) and females (p<0.001) ate fewer Kcals than their controls. In contrast, HFD increased total Kcals consumed in all groups with no effect of MD on this parameter.

**Figure 5:**
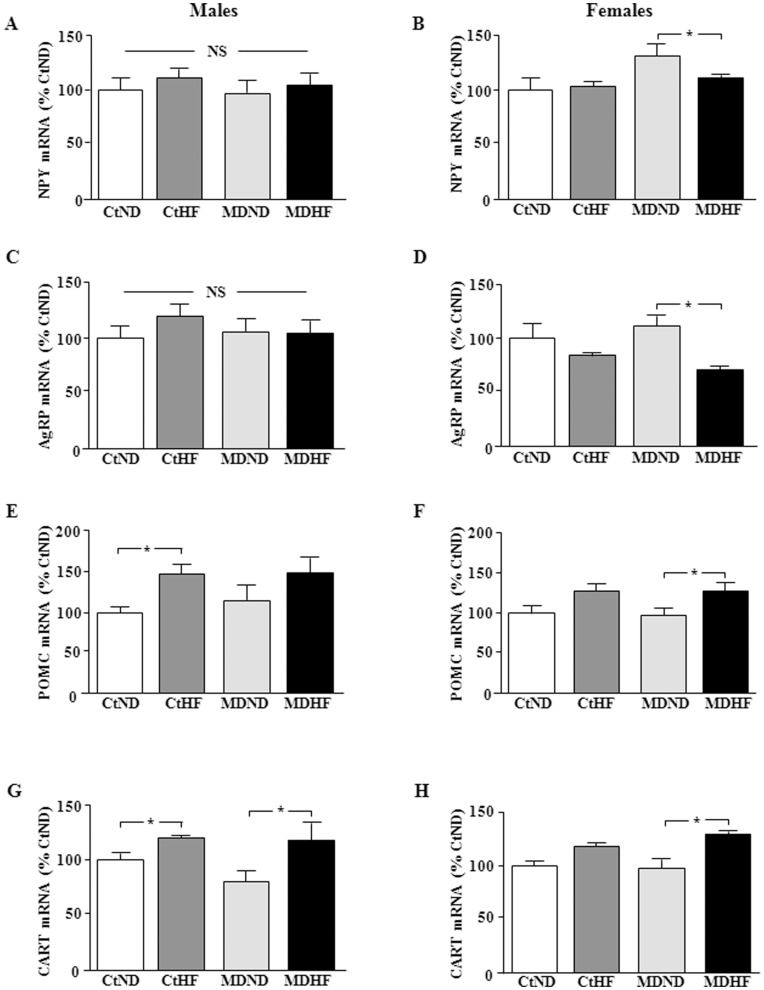
Hypothalamic mRNA levels of neuropeptide Y (NPY; A & B), Agouti related peptide (AgRP; C & D), proopiomelanocortin (POMC; E & F) and cocaine- and amphetamine regulated transcript (CART; G & H) in male (left column) and female (right column) rats. CtCD: controls on a control diet; CtHF: controls on a high fat diet from weaning onward; MDCD: maternally deprived rats on a control diet; MDHF: maternally deprived rats on a HFD from weaning onward. *  =  ANOVA p<0.05, NS  =  not significant.

### Serum Metabolic Factors

Levels of distinct metabolic factors at PNDs 35, 45, 65 and 85 are shown in Table 1. Levels at sacrifice are shown in Figure 4.

#### Glycemia

At PND35 glycemia was affected by sex, MD and diet (p<0.02). While in males glycemia was unaffected, in females there was an interaction between MD and diet (p<0.004). MD females had lower glycemia than controls and HFD increased glucose levels in female MD rats (p<0.02).

Glycemia was not affected by any of the experimental factors at PNDs 45 or 65. At PND85 HFD induced higher glycemia in control males compared to control females (p<0.04).

At sacrifice fasting glycemia was unaffected (males CtCD: 123.4±11.6, CtHF: 134.2±8.6, MDCD: 124.4±8.6, MDHF: 124.3±7.8; females CtCD: 132.0±9.8, CtHF: 122.6±7.5, MDCD: 127.9±5.7, MDHF: 118.9±5.5 mg/dl).

#### Insulin

There were no effects of any of the factors on insulin levels at PND35. At PND45 there was an effect of sex (p<0.0001) and diet (p<0.02), with an interaction between MD and diet (p<0.03). Males had higher insulin levels than females in all groups (p<0.0001) except controls on the HFD. In males insulin levels were affected by diet (p<0.03) with an interaction between MD and diet (p<0.04). HFD decreased insulin levels in control, but not MD males (p<0.03). At this age insulin levels in females were unaffected by MD or HFD intake.

**Figure 6:**
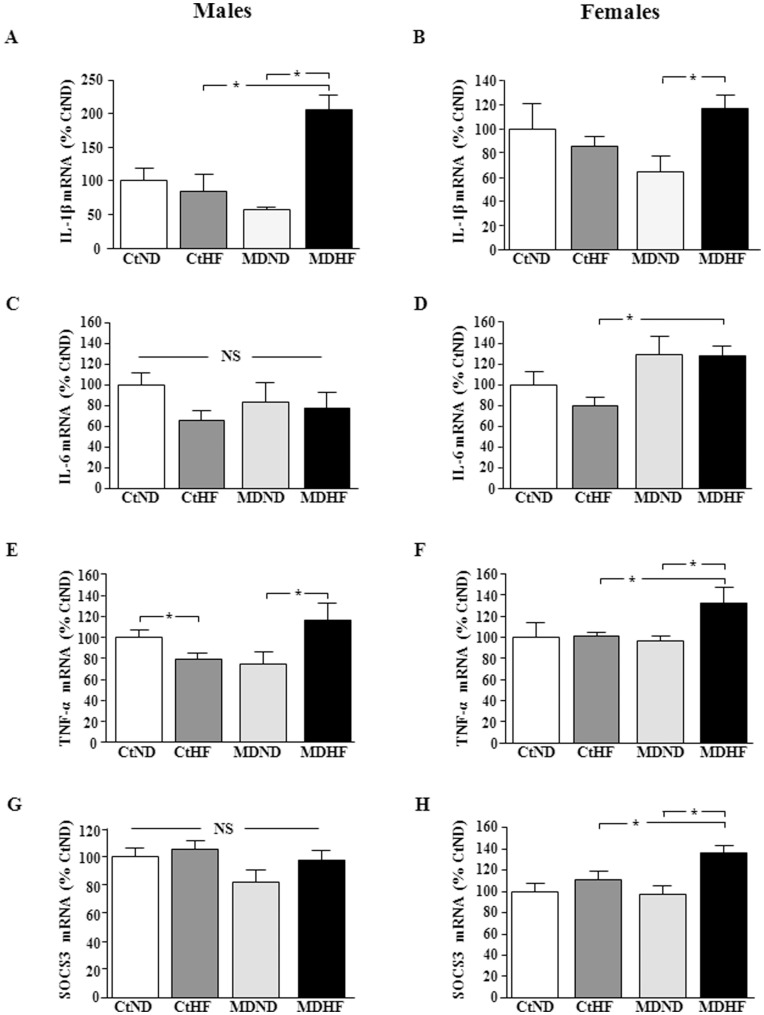
Hypothalamic mRNA levels of interleukin (IL)-1β (A & B), IL-6 (C & D), tumor necrosis factor (TNF)-α (E & F), and silencer of cytokine signaling 3 (SOCS3; G & H) in male (left column) and female (right column) rats. CtCD: controls on a control diet; CtHF: controls on a high fat diet from weaning onward; MDCD: maternally deprived rats on a control diet; MDHF: maternally deprived rats on a HFD from weaning onward. *  =  ANOVA p<0.05, NS  =  not significant.

At PND 65 insulin levels were affected by sex (p<0.0001), with males having higher levels than females in all groups (p<0.0001). Diet also affected insulin levels (p<0.0004), with HFD decreasing them in control males (p<0.05). In females insulin levels were affected by MD (p<0.05), decreasing them in rats on the control diet (p<0.002), and diet (p<0.0005) with HFD decreasing insulin levels in both control and MD females.

At PND85 insulin levels were affected by sex (p<0.0001), with levels being lower in females of all groups, and MD (p<0.02), with MD reducing insulin levels in males on a control diet (p<0.05).

At sacrifice serum insulin levels were affected by sex (p<0.0001) with males continuing to have higher levels than females in all corresponding groups (males CtCD: 2.7±0.2, CtHF: 2.6±0.3, MDCD: 2.3±0.3, MDHF: 4.0±0.9; females CtCD: 1.2±0.2, CtHF: 0.8±0.1, MDCD: 1.3±0.2, MDHF 1.3±0.3 ng/ml; p<0.0001). There was no effect of MD or diet.

#### HOMA-IR

There was an effect of sex (p<0.0001) on HOMA-IR at sacrifice (Fig. 4A), with males having a higher index in all corresponding groups (p<0.0001). Consumption of HFD increased the HOMA index in MD males, but not in any other group.

#### Triglycerides

At PND35 triglyceride levels were affected by MD (p<0.03), resulting in a significant reduction in females on a control diet. At PND45 triglyceride levels were modified by sex (p<0.0001) with males having higher levels in all corresponding group (p<0.0001), except for controls on a control diet.

There was no effect of any factor on triglyceride levels at PND65. At PND85 control males had higher triglycerides than females in response to the HFD (p<0.04).

At sacrifice triglyceride levels (Fig. 4B) were affected by diet (p<0.0001), decreasing in response to HFD in all groups (p<0.0005).

#### Leptin

At PND35 leptin levels were affected by MD (p<0.0001), with an interaction between MD and diet (p<0.005). MD rats of both sexes on a normal diet had decreased leptin levels (p<0.0001) and HFD increased leptin only in MD rats.

At PND45 leptin levels were influenced by MD (p<0.0001) and diet (p<0.0005) with interactions between sex and diet (p<0.05) and MD and diet (p<0.0002). Leptin levels were decreased in MD rats on a normal diet (p<0.0001). HFD increased leptin levels only in MD rats, with this increase being greater in males than in females.

At PND65 leptin levels continued to be affected by sex (p<0.0001) and diet (p<0.005), with interactions between sex and diet (p<0.04) and MD and diet (p<0.005). Males had higher levels than females in all groups (p<0.0001). On the control diet MD rats continued to have lower levels than their controls and HFD significantly increased leptin levels only in MD males.

At PND85 leptin levels were affected by sex (p<0.0001) and diet (p<0.0001), with interactions between sex and diet (p<0.0004) and MD and diet (p<0.003). Males had higher levels than females and MD rats continued to have lower levels than their controls (p<0.0001). In males HFD increased leptin levels in both controls and MD rats, while in females this increase was only seen in MD rats.

At sacrifice circulating leptin levels (Fig. 4C) were affected by sex (p<0.0001) and diet (p<0.0001), with interactions between sex and diet (p<0.0001), MD and diet (p<0.004) and sex, MD and diet (p<0.04). Levels were higher in males (p<0.0001) and MD rats on a control diet had decreased leptin levels. In males HFD increased leptin levels in both controls and MD rats, with this rise from control levels being greater in MD rats on the HFD. In females the increase due to HFD was only significant in MD rats.

#### Regression analysis

Insulin levels were significantly correlated with weight gain from weaning until the end of the study (R: 0.721; p<0.0001) and final weight (R: 0.733; p<0.0001). Leptin levels were also correlated with accumulated weight gain (R: 0.757; p<0.0001) and final weight (R: 0.759; p<0.0001). There was no relationship between weight gain or body weight with glycemia or triglyceride levels.

### Hypothalamic Gene Expression

Hypothalamic NPY mRNA levels were not affected by any of the experimental treatments in males (Fig. 5A), while HFD decreased them in female MD rats (p<0.03; Fig. 5B).

There was no effect of either MD or diet on AgRP mRNA levels in males (Fig. 5C), but in females they were affected by diet (p<0.006; Fig. 5D), with HFD decreasing AgRP in MD females (p<0.03).

In both males (p<0.03) and females (p<0.01) POMC levels were modified by diet (Fig. 5E and F, respectively). HFD increased POMC mRNA levels in all groups but this was only significant in control males and MD females.

Hypothalamic CART mRNA levels were affected by diet in males (p<0.02; Fig. 5G) and females (p<0.008; Fig. 5H), with HFD inducing CART levels in control males and MD males and females.

No effect on hypothalamic LepR levels was found (males CtCD: 100±8.9, CtHF: 92.9±10.1, MDCD: 97.1±17.2, MDHF: 123.0±12.1%; females CtCD: 100±9.2, CtHFD: 100.4±6.8, MDCD: 111.8±9.5, MDHF: 123.1±6.1%).

In males IL-1β levels (Fig. 6A) were influenced by diet (p<0.005) with an interaction between MD and diet (p<0.0007). HFD increased hypothalamic IL-1β levels in MD males, but not in controls. In females there was also an interaction between MD and diet (p<0.03), with HFD increasing IL-1β levels in MD females (Fig. 6B).

Hypothalamic IL-6 levels were unaffected in males (Fig. 6C) and increased by MD in females (p<0.0008; Fig. 6D), with this being significant in MD females on the HFD (p<0.04).

There was an interaction between MD and diet on TNF-α mRNA levels in males (p<0.02), with HFD decreasing them in controls and increasing them in MD rats (Fig. 6E). In females there was an effect of diet (p<0.05) with an interaction between MD and diet (p<0.05), as HFD increased TNF-α mRNA levels in MD females and had no effect in control females (Fig. 6F).

In males there was an interaction between MD and diet on GFAP mRNA levels (p<0.05), with HFD decreasing GFAP levels in controls and increase them in MD males, but these changes were not significant individually (CtCD: 100±9.5, CtHF: 77.9±5.1; MDCD: 67.3±6.0; MDHF: 81.5±9.8%). In females there was no effect of any treatment (CtCD: 100±10.1, CtHF: 110.0±10.3; MDCD: 102.2±14.0; MDHF: 123.2±6.0%).

There was no effect of any treatment on SOCS3 mRNA levels in males (Fig. 6G). In females HFD increased SOCS3 levels in MD rats (p<0.004; Fig. 6H).

## Discussion

Body weight and the propensity to become obese are determined by the interaction of an individual’s genetic make-up with environmental influences, including energy intake, dietary composition and energy expenditure, but also early environmental events. It is clear that prenatal and early postnatal occurrences such as stress, dietary inadequacies or hormonal modifications can have long-term implications in metabolism, modifying body weight and the response to later metabolic challenges [1]–[5], [8], [10], [17], [18]. We have previously reported that MD for 24 hours starting on PND9 reduces body weight and circulating leptin levels in a sex- and time-dependent manner [6], [15], [16], with the MD-induced weight reduction no longer being significant in early adulthood. Here we found this difference to persist at all stages, becoming more pronounced with advancing age in both males and females. One possible explanation for these differences is that the effect of MD on body weight is less pronounced in early adulthood such that a smaller sample size or even a slight increase in variability could result in no significant differences in some studies. Indeed, although in our previous study [16] the reduction in body weight did not reach significance in early adulthood, circulating leptin levels were significantly decreased. This suggests that these animals were metabolically affected. Furthermore, here we show that this early environmental assault renders these animals more susceptible to some of the negative effects of high fat intake.

The total energy consumed throughout the study by male MD rats on normal rat chow was significantly less than in control rats, which most likely contributes to their decreased weight gain. In contrast, during the last half of the study the mean daily energy consumption by MD females on normal chow was no longer different from their controls although they continued to gain less weight; thus, other mechanisms must be involved. This could include increased energy expenditure, but MD did not modify hypothalamic mRNA levels of orexigenic or anorexigeneic neuropeptides in either sex. This is in agreement with previous results where we found no effect of MD on hypothalamic mRNA levels of POMC, CART, NPY or AgRP levels at different post-natal ages [16].

It is conceivable that there are differences in the organization and functioning of the neurocircuits controlling appetite and metabolism that are not reflected in overall mRNA levels of these neuropeptides. MD affects hypothalamic cell-turnover, neurotrophic factors and markers of neuronal maturation during the separation period and up to 3 days later [15], [16], suggesting modifications in hypothalamic development. These changes could result from the decline in glycemia and circulating leptin levels and/or the rise in corticosterone observed in pups during the separation period [15]. Indeed, modifications in neonatal leptin levels induce long-term changes in metabolism [23]–[26] due, at least in part, to leptin’s neurotrophic effects on metabolic circuits [27]–[29]. Administration of a leptin antagonist at PND9, the age at which MD is induced here, results in hypothalamic changes at PND13 with females being more affected than males [30]. This pharmacological reduction in leptin’s actions neonatally is also associated with reduced weight gain in adult males [26]. Thus, the decline in leptin during MD probably induces some of the long-term metabolic effects reported here, but other factors are also likely to be involved. During MD pups not only experience food restriction and dehydration, but also thermal and psychological stress, all of which will contribute to the various metabolic, behavioral and psychological outcomes observed in this experimental model [31]. In humans maternal care during early stages of life is also associated with long-term consequences, including behavioral and metabolic outcomes [32]–[34], further emphasizing the importance of early life experiences.

The appearance of some long-term consequences of MD depends on the type of diet to which individuals are exposed. We found that weight gain and total Kcal intake were decreased in MD rats on a normal diet, but when exposed to a HFD the mean Kcal intake of these rats was not different from controls and their body weight increased to control levels. HFD has also been shown to reduce some psychological and behavioral effects of MD [17], [18], suggesting that HFD-induced modifications in behavior, stress and anxiety could also be involved in the observed changes in metabolism and weight gain.

In addition to decreasing leptin levels throughout development [6], [16] MD modified the HFD-induced changes in this metabolic hormone. Leptin levels rose significantly with HFD intake and weight gain as early as PND35 in MD rats of both sexes, while in control males they were not increased until PND85 and in control females leptin did not rise significantly even at PND102. Leptin levels were highly correlated with both accumulated weight gain and body weight, as previously reported for both sexes [35]. Not only do post-pubertal male rats have higher leptin levels than females, as seen here, but males are reported to have a greater relative increase in leptin concentrations per increase in body fat than do females [35], [36]. This, in conjunction with the relatively small rise in body weight in control females induced by HFD, could explain why no significant increase in leptin was found in these female rats.

In spite of the rapid rise in circulating leptin levels in response to HFD, MD rats continued to have increased energy intake. This suggests that central leptin sensitivity may be altered, although there was no effect of either MD or HFD on hypothalamic LepR mRNA levels, in agreement with earlier studies [16]. In contrast, during the period of maternal separation hypothalamic LepR mRNA levels are increased [15]. As hypothalamic metabolic circuits are developing at this time [37], long-term changes could be evoked in LepR distribution and/or the relative abundance of the different leptin receptor isoforms (*e.g.,* post-translational processing) that are not reflected in its mRNA levels. Moreover, leptin signaling can be affected without changes in receptor number, such as decreased intracellular leptin signaling due to an increase in SOCS3 [38]. The mRNA levels of this cytokine signaling inhibitor were unaffected in males, but increased in females in response to the combination of MD and HFD. This might suggest that females are more susceptible to developing leptin resistance and possibly other secondary complications in response to these factors. However, this was not reflected in the systemic metabolic parameters measured here.

HFD-induced hypothalamic inflammation is suggested to be involved in the development of obesity and its associated secondary complications, with inflammation occurring centrally before systemic inflammatory markers are detected and insulin and leptin resistance are incurred [20]. Here we found that HFD increased hypothalamic IL-1β and TNF-α mRNA levels in both male and female MD rats, but not in controls. Furthermore, this hypothalamic inflammatory state was associated with a rise in circulating leptin levels and HOMA index in male MD rats, but not in females. It is unknown whether these females would become insulin resistant after a longer period of HFD, but the fact that leptin levels rose rapidly (as early as PND35) in response to HFD and markers of hypothalamic inflammation were increased suggests that these processes do precede insulin resistance and possibly other secondary complications and in females these detrimental effects could appear after longer exposure to HFD. It should be taken into consideration that most studies reporting HFD-induced hypothalamic inflammation and its relationship with secondary effects have been performed in males and the mechanisms involved or the sensitivity to this phenomenon could differ in females. Indeed, although central inflammatory markers and SOCS3 were increased, HFD decreased orexigenic and increased anorexigenic neuropeptide mRNA levels in MD females. This would suggest that the increase in leptin levels continues to signal to these animals to decrease food intake and increase energy output, which could also be involved in impeding systemic changes such as increased insulin levels and HOMA-IR. Modulation of all four neuropeptides was not found in any other experimental group. These results are in agreement with other authors reporting that there is no change in neuropeptide mRNA levels in response to HFD or that the response is sex and time-dependant [39]–[41]. In addition, as reported here the early neonatal environment has previously been shown to affect the response to HFD [14], [42].

Our results indicate that MD may predispose rats to more rapid weight gain and development of secondary effects such as hyperleptinemia and hyperinsulinemia in response to HFD. Indeed, HFD did not induce hypothalamic cytokine production or increase the HOMA-IR in control rats, while MD rats developed hyperinsulinemia and hypothalamic inflammation in a sex dependent manner. Similar observations have been reported in obesity-prone (DIO) and obesity-resistant (DR) rats. Although HFD increases weight gain in both DIO and DR rats, DIO rats gain more weight and develop hyperleptinemia and hyperinsulinemia, while DR rats are hyperleptinemic, but normoinsulinemic [43]. The synaptic inputs to POMC and NPY/AgRP neurons in the hypothalamus of DIO and DR are significantly different, which is suggested to underlie their differential response to high fat intake [43]. Future experiments will be necessary to determine if MD induces organizational changes in the hypothalamus that changes their susceptibility to HFD, but it is clear that the metabolic response to high fat intake is determined by multiple factors.

Astrocytes have recently been suggested to play an important role in metabolic control [44]. These glial cells are reported to be activated in response to a HFD [20], [43], [45]; however, HFD did not modulate the mRNA levels of GFAP in either control or MD rats. Hypothalamic GFAP mRNA levels increase in response to high fat intake during adulthood [20], [43], [45]. It is possible that the astrocytic response is different when a HFD is introduced at weaning, as at this younger age the neuroendocrine circuits may be more capable of adjusting to this metabolic insult over time.

By the end of the study HFD decreased circulating triglyceride levels in both control and MD rats, with few changes at earlier ages. The effect of HFD on circulating triglyceride levels is variable in the literature and it is clear that the response is affected by starting triglyceride levels, other dietary components, metabolic status (*i.e*., insulin resistance or no) and time of day [46]–[50]. In addition, as these rats were fasted before sacrifice the decrease in triglycerides may reflect a differential response to fasting when on a HFD [51]. Circulating triglycerides can be derived from dietary fat or endogenous production and the removal of a large dietary source could result in a larger decrease in serum levels. A similar caveat must be taken into consideration when analyzing and interpreting all levels of circulating metabolic factors reported here. First, during development the results reported are non-fasting. In addition, when allowed to eat a HFD *ad libitum*, rodents modify their circadian patterns of eating and this is associated with decreased insulin and triglyceride levels with no change in glucose levels [50]. Whereas when HF feeding is restricted to normal eating patterns, although energy intake is similar, the metabolic changes are not [50].

The differential responses of males and females to MD and HFD are in accordance with previous reports as metabolic responses to early nutritional changes or stress [8], [10], [15], [16] and to HFD in adulthood [52], [53] are reported to be sexually dimorphic. Thus, it is clear that not only sex, but early life experiences and the interaction between these two factors can have significant effects on metabolism and the propensity to become obese. Not only should these concepts be taken into consideration when interpreting experimental results, but also in the search for effective treatments against the rapid rise in obesity and its secondary complications.
